# Dietary phytochemicals and cancer chemoprevention: a review of the clinical evidence

**DOI:** 10.18632/oncotarget.9593

**Published:** 2016-05-24

**Authors:** Ritesh Kotecha, Akiyoshi Takami, J. Luis Espinoza

**Affiliations:** ^1^ Department of Medicine, Beth Israel Deaconess Medical Center, Boston, MA, United States of America; ^2^ Department of Internal Medicine, Division of Hematology, Aichi Medical University, School of Medicine, Nagakute, Aichi, Japan; ^3^ Department of Hematology Oncology, Kanazawa University Graduate School of Medical Science, Kanazawa, Japan

**Keywords:** cancer chemoprevention, phytochemicals, resveratrol, curcumin, antioxidants

## Abstract

Cancer chemoprevention involves the use of different natural or biologic agents to inhibit or reverse tumor growth. Epidemiological and pre-clinical data suggest that various natural phytochemicals and dietary compounds possess chemopreventive properties, and in-vitro and animal studies support that these compounds may modulate signaling pathways involved in cell proliferation and apoptosis in transformed cells, enhance the host immune system and sensitize malignant cells to cytotoxic agents. Despite promising results from experimental studies, only a limited number of these compounds have been tested in clinical trials and have shown variable results. In this review, we summarize the data regarding select phytochemicals including curcumin, resveratrol, lycopene, folates and tea polyphenols with emphasis on the clinical evidence supporting the efficacy of these compounds in high-risk populations.

## INTRODUCTION

Despite modern advances in medical therapeutics worldwide, cancer continues to account for more than fourteen million new cases and roughly eight million deaths each year [1]. Increases in global cancer incidence over the past several years have led to campaigns focused specifically on disease prevention, and detectable declines in global cancer incidence have been achieved through efforts such as tobacco cessation movements and vaccinations [1]. Epidemiological studies have also uncovered that diet and exercise may significantly impact the prevalence of specific types of cancers, renewing interest in dietary phytochemical research [2–4].

Phytochemicals constitute a heterogeneous set of bioactive compounds classified by chemical structure and include polyphenols, alkaloids, carotenoids, and nitrogen compounds [5]. These compounds are naturally found in fruits, vegetables, grains and other plant products and are often responsible for distinct plant characteristics such as color pigmentation and smell. Moreover, many are integral for host protection against viruses, parasites and other externally damaging agents. Initial studies have revealed that these compounds are able to affect cell proliferation and cell cycle regulation, and usually participate in multiple signaling pathways which are often disrupted in tumor initiation, proliferation and propagation [5–9].

Although prior observations have guided multiple successful pre-clinical studies, only a limited number of clinical trials have been able to fully expose the distinct impact each dietary phytochemical may have on cancer prevention [8]. Many of these failures have been attributed to the variable bioavailability and distribution of compounds, optimal mixtures of several phytochemicals, and the appreciable risk reduction that may take several years to detect in large population studies. As global cancer incidence continues to rise, understanding the impact of these dietary modifications may fuel simple and inexpensive ways to improve health worldwide. Here, we review the history and latest clinical findings on select dietary compounds including curcumin, resveratrol, tea polyphenols, lycopene and antioxidants.

## AN OVERVIEW OF CARCINOGENESIS

Carcinogenesis is a multistep process characterized by a progression of distinct molecular changes that ultimately reprogram and transform a cell to undergo uncontrolled cellular division [10]. During the last fifty years, research has uncovered innumerable critical molecular players and targeted pathways, and highlighted the underlying balance of aberrant activation of proto-oncogenes and inactivation of tumor suppressor genes. With each disruption, cells undergo changes fundamentally represented by tumor initiation, promotion and progression [11, 12]

Tumor initiation is a rapid and irreversible process that starts with an exposure to a carcinogenic agent, followed by its distribution and transportation to tissues causing non-lethal mutations in cellular DNA. These “initiated cells” begin to accumulate additional irreversible genetic changes which persist with each new cycle of proliferation [12]. Functionally, initiated cells are more immune to inhibitory signals mediated by cell differentiation inducers and negative growth regulators [13, 14].

Tumor promotion involves the selective clonal expansion and proliferation of initiated cells allowing for additional mutations to accumulate. In contrast to initiation, tumor promotion is a relatively lengthy and reversible process in which actively proliferating pre-neoplastic cells begin to divide and propagate. Tumor progression, the final stage of neoplastic transformation, occurs after these mutations result in an invasive cellular phenotype with metastatic potential [12, 15].

Advances in our understandings of tumor development show that each step is composed of highly variable and intricate systems. For instance, epigenetic changes of tumor suppressor genes through DNA methylation in pre-neoplastic tissues may result in accelerated carcinogenesis [16, 17]. The dynamic regulation of proteins involved in cellular apoptosis by micro-RNAs may significantly impact both tumor promotion and progression [18]. Finally, more recent evidence has highlighted the critical role of the tumor microenvironment on the survival and mutation of pre-neoplastic cells [14].

**Figure 1:**
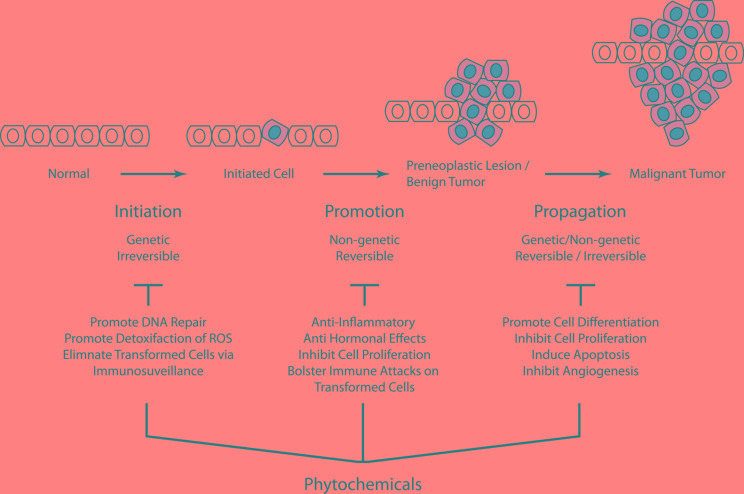
Carcinogenesis is a multistep process that ultimately reprogram a normal cell into a cancer cell Phytochemicals may exert their chemopreventive effects by blocking key events of tumor initiation and promotion thus reversing the premalignant stage. These agents may also prevent tumorigenesis by inhibiting or retarding tumor progression or by promoting cell differentiation.

Cancer chemoprevention centers on the identification of agents that specifically impact early stages of cellular transformation [19, 20]. Naturally occurring phytochemicals have been found to have a wide range of cellular effects (Figure 1). For instance, phytochemicals may prevent carcinogens from reaching targeted sites and support detoxification of highly reactive molecules [21]. Select phytochemicals also enhance innate immune surveillance and improve the elimination of transformed cells [22]. Finally, phytochemicals have several impacts on intrinsic DNA repair mechanisms and may influence tumor suppressors and inhibit cellular proliferation pathways [19].

## CURCUMIN

Curcumin, or turmeric (bis-α, β-unsaturated β−diketone), is a polyphenol derived from the roots of the perennial *Curcuma Longa* plant, and is a gold-colored spice widely used in Indian cooking, textile dyes, and in traditional Ayurvedic medicine [23]. In recent decades, *in vitro* models have shown that curcumin inhibits the growth of a variety of cell lines by inducing cell cycle arrest and apoptosis, most importantly through pleiotropic modulation on several distinct cancer targets including nuclear factor kappa B (NF-κB), cyclooxygenase-2 (COX-2), tumor necrosis factor alpha (TNF-α), STAT-3 and cyclin D1 [23–26]. Building upon pre-clinical work, several phase I clinical trials have confirmed both the safety and pharmacokinetics of curcumin in patients with doses escalated up to eight grams per day, and these trials have shown measurable biological effects in patients with a variety of malignancies including pancreatic cancer, multiple myeloma and advanced colorectal cancer refractory to standard chemotherapy [27–30].

Curcumin is preferentially distributed into the colonic mucosa compared with other tissues, leading many initial clinical studies to focus on identifying whether this compound may play a role in colorectal cancer models [24]. This hypothesis was tested in patients with familial adenomatous polyposis (FAP), an inherited condition that leads to unregulated development of innumerable pre-cancerous adenomatous growths throughout the colon with eventual development of colorectal cancer at a young age. In one study, patients were given a combination of curcumin and quercetin (400/20 mg), a common flavonoid compound found in several supplements and foods. Compared to baseline colonoscopies performed prior to initiation of treatment, all five patients tested were found to have a decreased number of polyps and reduced polyp size after six months of treatment without other laboratory abnormalities and minimal adverse effects [31]. Although this study was limited and used a combination of both curcumin and quercetin, it raised awareness for future research testing the therapeutic potential of curcumin in pre-cancerous models [31].

Other small studies have similarly shown that high doses of curcumin may have a preventive effect in pre-cancerous models. In an open-label phase II clinical study, Carroll and colleagues investigated the effects of daily curcumin on aberrant crypt foci (ACF). ACF are proposed to be the earliest histologic sign for colonic neoplastic lesions and are composed of abnormal crypt zones with high levels of prostaglandin-E2 (PGE-2) and 5-eleicosatetraenoic acid (5-HETE). Participants were randomized to either two or four grams of daily curcumin, and underwent colonoscopies prior to and after thirty days of treatment [32]. Although this study found no significant reduction in PGE-2 or 5-HETE serum levels, patients taking four grams of daily curcumin were found to have a 40% reduction in the number of ACF lesions compared with those taking two grams of curcumin. Moreover, a recent phase I clinical trial highlighted that these clinical effects may be applicable in other high-risk precursor lesions including oral leukoplakia, intestinal metaplasia of the stomach and cervical intraepithelial neoplasia, as some treated patients were found to have histological improvement of precursor lesions compared to baseline after three months of high dose curcumin supplementation [33].

To extend the potential clinical effect of curcumin in these high-risk populations, current research has shifted focus to improving bioavailability to overcome both the variability of absorption and rapid compound metabolism. Several attempts combining curcumin with glucuronidation inhibitors such as piperine to inhibit hepatic and intestinal metabolism have shown promising results [34]. Other efforts have focused on altering compound delivery by liposomal or phospholipid complexing, as well as utilizing curcumin analogues and nanoparticles [35, 36]. Recently, a phase I dose-escalation study showed that participants receiving single dose liposomal curcumin (10-400 mg/m^2^) had a dose dependent increase in both the plasma concentration of curcumin and its active metabolite tetrahydrocurcumin (THC) without clinical side effects. Red blood cell morphology changes were seen at curcumin doses greater than 120 mg/m^2^, possibly indicating a dose limiting sign of toxicity [37]. Additionally, liquid micellar curcumin formulations have shown to have significant bioavailability without increased toxicities. In a recent crossover study, patients given liquid micellar formulations compared to curcuminoid powder or micronized powder had a 185 fold increase in bioavailability within twenty four hours, with a two-fold increase in women compared with men in absorptive efficacy [38].

## RESVERATROL

Resveratrol is a phytoalexin found in many fruits and plants including red wine, grapes, berries and peanuts [39]. The roots of the p*olygonum cuspidatum*, or Japanese knotweed, contains the highest naturally occurring levels of resveratrol and has been used in traditional Japanese and Chinese medicinal treatments for dermatitis, bacterial infections and inflammation. Plants produce resveratrol in response usually to mechanical injuries, ultraviolet radiation and as a defense for viral and fungal infections [39, 40]. Resveratrol has become the most common phytoalexin currently studied in modern healthcare because of the highly publicized “French Paradox”, an observation that there is a relatively low incidence of cardiovascular disease in France despite a particularly rich saturated-fat diet from the high consumption of red wine [41].

Early studies identified that resveratrol has anti-cancer effects against several different tumor type and affects multiple stages of tumor initiation and proliferation. Specifically, resveratrol can induce cancer cell apoptosis by interfering with multiple signaling pathways activated in transformed cells [40–45]. Clinical trials have also defined the safety, pharmacokinetics and metabolism as both a single synthetic agent and constituent of food at variable doses [46]. These studies also showed that adverse effects including diarrhea, nausea and abdominal pain occur in those taking more than one gram of resveratrol daily, which has now become a standard dose limit in subsequent clinical work [46].

Resveratrol supplementation and its potential effects in healthy subjects have been recently tested in clinical trials. Firstly, one study investigated the effects of resveratrol at variable high doses (0.5, 1, 2.5 and 5 grams / day for 29 days; *n* = 10-12 / dose) on circulating levels of insulin-like growth factor (IGF-1) and IGF-binding protein 3 (IGFBP-3) [47], two markers often associated with increased tumor formation and subsequent metastasis [48]. Unexpectedly, these doses were found to be safe and higher doses of five grams daily were associated with mild gastrointestinal adverse effects. Subjects given 2.5 grams daily of resveratrol showed significantly reduced levels of circulating IGF-1 and IGFBP-3 plasma levels [47], suggesting possible chemopreventive potential. Future analyses using these surrogates will need to be interpreted with caution as concentrations of IGF-1 and IGFBP-3 vary in different cancer models and are strongly influenced by other dietary compounds including citrus fruits and Vitamin C [49].

Chow and colleagues set out to investigate whether resveratrol would have an effect on drug metabolism and enzymes associated with carcinogenesis. In their study, one gram of resveratrol daily for four weeks was found to have significant inhibitory effects on plasma cytochrome P450 enzymes, including CYP3A4, 2D6, and 2C9, and enhanced CYP1A2 compared with baseline measurements in healthy volunteers [50]. These modulatory effects on enzymatic systems used in detoxification and carcinogen activation may account for some chemopreventive effects, and also importantly may alter metabolism of other agents. As many chemotherapeutic and other medications are metabolized through the cytochrome system, this study critically raises safety concerns for co-administration with other pharmaceutical agents.

The effect of resveratrol has also been heavily studied in patients with breast cancer. In a case-control study, Levi and colleagues showed that women with high total intake of resveratrol had a lower risk of breast cancer compared with women with a low level of ingestion (OR: 0.39) [51]. More recently, a randomized, double-blind placebo trial showed that in women at an increased risk for development of breast cancer, twice daily resveratrol dosing for twelve weeks was associated with a decrease in methylation of four cancer related genes on mammary tissue biopsies post-treatment. This work highlights the direct in-vitro anti-proliferative effect and mechanism in tissue specimens after treatment [52]. Other pre-clinical and clinical studies have proposed that resveratrol may also modulate hormonal metabolism and effects used in breast cancer and prostate cancer. In a randomized placebo controlled trial, Kjaer and colleagues showed that although resveratrol intake was not associated with prostate size or reduction in prostate serum antigen levels, it was associated with lower levels of androgen precursors including androstenolone (DHEA) and therefore may have a relevant effect in benign prostatic hyperplasia and cancer growth [53]. These findings are consistent with previously pre-clinical work reporting that resveratrol suppresses prostate cancer growth in rat models, an effect largely mediated through down-regulation of androgen receptor expression and suppression of androgen responsive glandular kallikrein, an orthologue of the human prostate specific antigen (PSA) [54].

The potential therapeutic effect of resveratrol may also be through promoting immunosurveillance through the innate immune system thereby enhancing elimination of spontaneous tumor cells prior to proliferation. Natural killer (NK) cells are the primary effector lymphocytes of this system and are able to importantly recognize transformed or infected cells without prior education by antigen process cells. This allows NK cells to effectively eliminate rapidly progressing tumor cells at a much more rapid rate compared with T lymphocytes, which require antigen recognition and education prior to activation [55]. NKG2D, an antigen receptor expressed by cytotoxic lymphocytes including NK cells, CD8 and γδ T cells, appears to play a significant role in tumor surveillance as these cells utilize the NKG2D receptor to identify specific surface ligands expressed on transformed cells for cytotoxicity [56].

To delineate the potential role of resveratrol in the innate immune system, a clinical trial focused on detecting differences in immune system profiles was performed in healthy subjects given one gram of resveratrol daily for two weeks. Administration of this compound was found to correlate with enhanced expression of NKG2D receptor on circulating peripheral blood NK cells [57]. Since pre-clinical studies have confirmed that resveratrol can induce the expression of NKG2D ligands in transformed cells and thus render these cells more susceptible to NK cell lysis *via* NKG2D cytotoxic pathways [22], this study suggested that resveratrol may modulate this axis to allow for increased tumor surveillance by the innate immune system [22].

Pharmacokinetic evidence indicate that resveratrol has poor bioavailability due to its rapid and extensive liver metabolism which severely impairs its therapeutic effects [58, 59]. Several approaches, with variable results, have been attempted to overcome this problem [60], including combining resveratrol with glucuronidation inhibitors such as piperine [59], developing resveratrol nanoparticles [61, 62], and utilizing novel drug delivery systems to protect and stabilize resveratrol to enhance its bioavailability [63, 64]. Further studies in humans are needed to determine the optimal delivery system to achieve clinically relevant levels of resveratrol.

## TEA POLYPHENOLS

*Camellia sinensis*, or tea, is one of the most ancient and popular beverages consumed across the world. Although the specific composition varies widely, tea is usually composed of a combination of polyphenols, alkaloids, minerals, and other volatile organic compounds [65]. Further, there is a very high proportion of catechin polyphenols such as Epigallocatechin-3-gallate (EGCG) and Epicatechin-3-gallate (ECG). Interestingly, green tea contains much higher concentration of these specific catechins compared with other black teas [66]. Catechin polyphenols, particularly EGCG and EBCG have robust antioxidant activity and are thought to exert their role as antioxidants by preventing specific DNA damage by reactive oxygen species, thereby preventing tumor mutagenesis of normal cells [65, 66]. In pre-clinical studies, tea polyphenols have been shown to directly inhibit tumor cell growth by inducing apoptosis through multiple pathways linked in cancer development [67]. Tea polyphenols have also been implicated in multiple carcinogenesis pathways including inhibiting angiogenesis modulating the immune system, and activating enzyme systems involved in cellular detoxification through the glutathione S-transferase and quinone reductase pathways [68].

Multiple studies have shown overall conflicting data regarding the potential cancer risk reductive properties of green tea in various populations [69]. This inconsistency in results may be in part because multiple types of teas are often used in trials and have variable tea preparations, unknown concentrations of different types of antioxidants, and the bioavailability of many of these compounds after ingestion is different across populations. Furthermore, many of these studies are often confounded by other ingestions that may overall lead to the development of cancers including tobacco and alcohol use and it is difficult to distinguish these confounding variables [70].

To date, there have been several clinical trials that have set out to identify the potential clinical role these tea polyphenols may have in cancer prevention. Two randomized clinical trials evaluated the effects of tea extracts on premalignant oral lesions called leukoplakia. In a double blinded interventional trial, subjects were given either three grams of mixed tea product, both orally and topically, or placebo. After six months of treatment, 38% of patients in the treatment group had partial regression of their oral lesions compared to 10% in the placebo group. Furthermore, progression in the lesions size was lower compared to the treatment group, 3% vs. 7% [71]. In addition, a second trial repeated this with pre-malignant oral lesions randomly assigned to receive either 500 mg, 700 mg or 1000 mg/m^2^ of tea extract compared to placebo three times daily for twelve weeks. Although not statistically significant, those in the tea extract arm were found to have an overall better clinical response and improvement in lesion histology [53].

Other studies have utilized the impact of treatments on specific biomarkers as potential signs for cancer risk reduction. Urinary levels of 8-hydroxydeoxygnaunosine (8-OHdG), a biomarker of oxidative DNA damage has been shown to be elevated in patients with lung, liver, kidney, brain, stomach and ovarian cancers [72]. Recently, randomized controlled clinical trials showed that green tea significantly decreased urinary levels of 8-OHdG in adult heavy smokers [73, 74]. These findings were confirmed in another trial, as high risk individuals for liver cancer due to hepatic infections or aflatoxin was given green tea supplements of 500 or 1000 mg daily for 3 months compared with placebo showed significantly lower 8-OHdG levels [75]. In contrast, levels of pepsinogen, a marker of gastric atrophy that has been shown to indicate increased risk for stomach cancer, were not affected by treatments with tea polyphenols for one year of treatment [76]. Therefore, although tea polyphenols have shown to influence certain biomarkers involved in cancer propagation, the clinical risk reductive effect of polyphenols still remains unclear. This conclusion was further validated after Zheng and colleagues in a study of prostate cancer in the Asian population [59].

## ANTIOXIDANTS

Reactive oxygen species (ROS), or free radicals like superoxide (O2−), hydrogen peroxide (H2O2) and peroxynitrite (OONO−) are produced during aerobic cellular metabolism. In normal cells, low level concentrations of these compounds are required for signal transduction, however excessive levels of ROS can induce damage to all cellular components, including proteins, lipids, carbohydrates, and nucleic acids [77]. Oxidative stress reflects an imbalance between production of ROS and an adequate antioxidant defense. Due to the unchecked dramatic cell proliferation of cancer cells, higher amounts of ROS are produced with increased proliferation [77, 78].

Antioxidants are known as free radical scavengers since they are able to interact with and neutralize free radical species. Endogenous antioxidants naturally produced in the body help neutralize these ROS, and external sources of antioxidants are also supplanted from fruits, vegetables and grains [11]. Lycopene, beta carotene, Vitamins A, C, E, selenium and other dietary antioxidants have been broadly studied in humans for preventing inflammation, cancer and other stress related disease. Pre-clinical studies have shown that antioxidants are capable of preventing cellular damage induced by free ROS, suggesting that cancer development may be slowed in the setting of increased levels of dietary exogenous or endogenous antioxidant supplements [79].

Epidemiological studies have shown mixed results regarding antioxidant supplementation and effects on primary cancer prevention [80]. One of the most prominent first trials was the Carotene and Retinol Efficacy (CARET) which examined the effects of daily supplementation with beta carotene and retinol on the incidence of lung cancer, and other cancers and death from incidence, and showed that both beta carotene (15 mg) and retinol (25,000 IU) daily supplementation was associated with increased lung cancer and increased all-cause mortality [81]. These adverse effects persisted up to six years after supplementation ended as reported in an updated study with the caveat that the higher risk of lung cancer and all-cause mortality were no longer statistically significant [82]. Similarly, the Linxian trial showed that a combination of 15 mg of beta carotene, alpha tocopherol 30 mg, and selenium 50 μg daily for five years initially showed a lower mortality risk from gastric cancer but not esophageal cancer. The study also concluded that polyphenols did not affect the risk of developing either gastric or esophageal cancer [83]. A new report ten years later analyzing those who took this antioxidant supplementation compared with placebo failed to show this persistent reduced risk of mortality [84].

Trials that have failed to show clinical significance of antioxidant therapy in cancer prevention have been performed in a variety of other models. Beta-carotene and/or alpha-tocopherol has failed to show an effect on the incidence of lung cancer and other cancers including urothelial, pancreatic, colorectal, and digestive tract cancers [85–88]. Expanding this to other supplements has also yielded mixed results. Clinical studies of alpha tocopherol (400 IU) and/or Vitamin C (500 mg) in combination *versus* placebo did not reduce the incidence of prostate cancer or other cancers including lymphoma, leukemia, melanoma, lung, bladder pancreas, or colorectal cancers in male U.S physicians older than 50 years of age for a median of 7.6 years of follow-up [89]. The authors also concluded post-trial that after a mean of 10.3 years of follow-up, alpha-tocopherol and Vitamin C supplementation had no immediate or long-term detectable effects on the risk of total or site-specific cancers [90].

There have been several studies that have highlighted that although anti-oxidants may have clinical roles in cancer pathways, there is still much to be understood about combinations to yield positive results. The *“Supplementation en Vitamins et Meraux Antioxidants”* (SU.VI.MAX) trial utilized a combination of several antioxidants and minerals including Vitamin C (120 mg), Vitamin E (30 mg), beta-carotene (6 mg), selenium (100 μg) and Zinc (20 mg) showed no global effect on cancer, cardiovascular disease, or all-cause mortality during median 7.5 years of treatment. However, specific analyses revealed an increase in skin cancer incidence among women only, leading to an overall lower cancer incidence and mortality among men only [91]. An updated study of these patients revealed that the effect disappeared within 5 years of ending supplementation [92].

A similar study showed that a combination of selenium (200 μg) and Vitamin E (400 IU) daily for a median of 5.5 years did not reduce the incidence of prostate or other cancers in men older than 50 [93]. However, updated findings from this study showed that after a mean of seven years, the incidence of prostate cancer was 17% higher among men taking Vitamin E alone compared with men taking placebo [94]. This adverse effect was not observed in the selenium alone or Vitamin E and selenium groups [93]. Selenium supplementation has recently been shown to have no clinical benefit in men with low selenium baseline levels, and instead increases the risk for prostate cancer in those who have high baseline selenium levels [95]. Moreover, Vitamin E supplementation also was found to have an increased risk of cancer in those who have low selenium status, and therefore authors have concluded that men should avoid selenium or Vitamin E supplementing at higher than recommended doses [96].

One possible reason for the lack of positive clinical results in antioxidant chemo preventive therapy can be that many antioxidants are consumed in a complex mixture of antioxidants, vitamins, and minerals. Differences in chemical composition of naturally occurring antioxidants in food compared with those purified into supplements may contribute to these effects. For instance, Vitamin E, which can be found in eight different chemical forms in nature and usually found in supplements as alpha-tocopherol [97]. Additionally, although many of these studies have been performed over long time periods, the executive effect of antioxidants may require longer longitudinal studies to identify small effects, and may only apply to individuals who already have increased oxidative ROS species at baseline. Therefore, further studies that identify balanced composites of vitamins, antioxidants and minerals to create a balanced combination and applying this to individuals with measurable increased oxidative stress may help yield more encouraging results.

## FOLATE AND FOLIC ACID

Folate, also known as folacin, pteroylglutamic acid or vitamin B9, is a water-solute B-vitamin that is a cofactor in carbon transfer reactions essential in DNA synthesis, repair and methylation [98]. Since humans are incapable of synthesizing folates *de novo*, many supplements and foods are fortified with a synthetic form called folic acid. Important dietary sources include green leafy vegetables, asparagus, and broccoli [99].

Preclinical studies have suggested that folate may have anti-cancer properties because of its role in DNA repair and its role in modulating S-adenosylmethionine, a universal methyl donor group for DNA methylation reactions. Therefore, several large scale cross sectional studies have shown that dietary folate intake may be associated with a lower risk of several cancers including lung, breast, pancreatic, esophagus, stomach and colorectal cancer [100, 101]. Results from large prospective studies have shown that there is a near 25% risk reduction in the risk of colorectal cancer in those with high folate intake compared with low intake. Recently, a meta-analysis (16 prospective and 26 case control studies) revealed that women with higher daily dietary folate intake had a significant reduction in breast cancer risk compared with those with lower folate intake. Interestingly, despite this effect, there was no significant association between circulating folate levels and breast cancer risk [102].

Building upon epidemiologic population data, several clinical trials have been conducted to assess whether folic acid supplementation is associated directly with appreciable cancer reduction. In a small placebo controlled trial in which 94 patients with colorectal adenomas were assigned to receive either a daily 5 mg dose of folic acid or placebo, folic acid supplementation was associated with a 3-fold decrease in colonic polyp recurrence at three year follow-up [103], however larger trials and meta-analyses have failed to show a reduction in colorectal adenoma risk [104, 105]. The unexpected results from these studies have raised the possibility that folic acid supplementation may actually increase the risk of colorectal neoplasia [106]. For instance, a recent meta-analysis from Wien and colleagues evaluated twelve randomized controlled trials and seven observational studies and concluded that there was no difference in cancer incidence between controls and participants taking folic acid supplements in observational studies. However, meta-analyses of the randomized controlled trials showed a modest increase in frequency of overall cancer in the folic acid groups with a relative risk of 1.07 (95% CI: 1.00-1.14). Notably, further examination showed that prostate cancer was the only cancer type with a significant increase in risk associated with folic acid supplementation [107].

## LYCOPENE

Lycopene is a naturally occurring carotenoid found in many fruits and vegetables, with particularly high concentration in tomatoes and tomato-based products [108]. Experimental studies have shown that lycopene lowers intracellular generation of reactive oxygen species (ROS) by possibly augmenting proteins involved in antioxidant reactions including superoxide dismutase-1 (SOD-1) and glutathione-S-transferase-omega-1. Lycopene may also reduce oxidative stress by down-regulating expression of ROS generating proteins such as ERO-1 like protein-α and CLIC-1 [109]. Furthermore, lycopene has also been shown to inhibit cell proliferation, induce apoptosis, and in prostate cancer models, has shown to attenuate the metastatic capacity of cancer cells [109, 110].

Consistent with prior experimental data, epidemiological and observational studies have linked increased consumption of lycopene-rich food with lower prostate cancer risks [111–113]. In a meta-analysis of twenty-one observational studies, both moderate and high lycopene rich diets were associated with lower prostate cancer incidence, 6% and 11% respectively. Though this study concluded that the trend was not statistically significant, it highlighted that single interventional randomized trials are required to assess the true clinical effect [114]. A recent meta-analysis of eight randomized clinical trials showed a minor, insignificant decrease in the incidence of benign prostatic hyperplasia and prostate cancer patients compared to controls [112].

Interestingly, a recent double blinded randomized controlled trial focused on patients with high-grade prostatic intraepithelial neoplasia and/or atypical small acinar proliferation. Patients in this trial were given a high dose supplement containing lycopene, selenium and green tea catechin for six months, and showed that there were no significant difference in PSA levels between the two groups, but there was a higher incidence of prostate cancer at re-biopsy. Further, micro-RNA profiling of the biopsy samples had higher levels of prostate cancer specific progression biomarkers, thereby concluding that high doses of these types of supplements should be avoided in patients with prostatic intraepithelial neoplasia [115]. Other clinical studies have shown similar results that lycopene correlates with a significant decrease in PSA levels, however the significance has not been mirrored in clinical outcomes [112, 113]. With the limited number and heterogeneity of existing studies, there is insufficient evidence to support or refute the use of lycopene in pre-cancer models.

## ADDRESSING CHALLENGES FOR FUTURE CLINICAL TRIALS

Despite the potential public health benefit and scientific importance, cancer chemoprevention has not been widely adopted in clinical practice. The poor translational of many pre-clinical findings into clinically effective cancer preventative therapies may be in part due to limitations of current clinical trial design, and failure to identify those specifically at high risk. Changing the way trials are performed in the future may help uncover potential health benefits in significant ways.

Cancer incidence is commonly used as a primary endpoint in many clinical trials to evaluate the impact of an intervention on cancer prevention. As described earlier, the latency from progression of malignant cells transformations to detectable cancer lesions may sometimes require decades of patient data. Many studies may lack the financial budget or research infrastructure to overcome this practical obstacle, and adherence to a prescribed regimen may continue to decline as trials progress [7, 116, 117]. Utilizing high-risk pre-malignant lesions including adenomas, colonic ACFs, mammographic breast densities, or intraepithelial neoplasia in the head and neck as surrogates may also influence the predictive accuracy of therapies as many lesions have variable frequencies for transformation and spontaneous regression [118, 119].

Utilizing cancer chemical biomarkers may represent a better way to assess responses in patients taking these dietary phytochemicals. In a chemoprevention setting, an optimal cancer biomarkers should be accurately measurable and occur prior to cancer development and correlate with malignant transformation and progression [117]. High-throughput screening methods utilizing functional genomic, transcriptomics, proteomics and metabolomics studies have identified many new potential biomarkers of early carcinogenesis. For instance, genomic profiling of airway epithelial samples from smokers at high risk for lung cancer have identified a genomic signature compatible with the phosphatidylinositol 3-kinase (PI3K) pathway activation in cytologically normal bronchial airways of smokers with lung cancer and dysplastic lesions, suggesting that PI3K activation in the proximal airway is a measurable, reversible step preceding development of lung cancer [120]. Genomic studies have also identified miRNA and target gene dysregulation in other precancerous lesions, supporting that measurement of miRNAs may also represent potential biomarkers for cancer detection [121, 122]. In addition, plasma metabolomics studies have shown distinct glucose metabolism profiles during progression from chronic atrophic gastritis to intestinal metaplasia, gastric dysplasia and ultimately to gastric cancer [123]. Similar unique metabolic patterns have been identified in lung cancer [124] and oral squamous cell carcinoma pathways [125] as well. By incorporating a more integrative approach, Bro and colleagues recent created *Biocontour* [126], a novel risk assessment scale that combines metabolic profile analysis of plasma samples and relevant lifestyle information to predict cancer risk. With a sensitivity and specificity superior to traditional screening mammography, *Biocontour* was shown to predict individual diagnoses of breast cancer several years ahead of diagnosis [126]. If these applications are validated in larger populations, future studies may be able to implement these tools to predict the effect of interventions on cancer development and help identify those individuals with high-risk that should be enrolled in cancer prevention trials.

A fundamental challenge that still remains in dietary phytochemical research is the lack of consensus regarding the optimal dose of many of these compounds to be used in trials. High doses of synthetic bioactive agents have been commonly administered based frequently on data obtained in pre-clinical studies, and likely represent unrealistic and non-physiological conditions. It is conceivable, though, that phytochemicals exert their optimal anti-cancer activity at dietary relevant doses. Cai and colleagues compared the target-tissue distribution and activity of low dietary doses (5 mg) with an intake 200 fold higher, and found that low dietary dose not only elicit biological changes in mouse and human colonic tissues, but also have superior efficacy compared to higher doses [127]. In this study, low concentrations of resveratrol prevented colonic tumor progression in mice and correlated with the induction of AMPK and senescence, and these effects were also notably reproduced in human tissue. These results may indicate that other diet-derived agents may exert cancer chemopreventive qualities at low dietary doses as well.

## CONCLUDING REMARKS

Over the last two decades, there have been several studies that have clearly shown that dietary agents have anti-cancer properties and epidemiological studies have corroborated that cancer prevalence varies based upon several factors including dietary consumptions. Despite encouraging in-vitro data, randomized clinical trials aimed at exposing these effects have been extremely difficult to characterize. To improve upon the ability to identify these effects, many studies have identified predictors including biomarkers and other surrogate markers such as high risk pre-cancerous lesions to serve as additional predictors to identify regression and progression as markers of change. Understanding how these dietary agents interact with cancer cells, the immune system and oxidative stress pathways may uncover safe, non-toxic and economical anti-cancer therapeutics in the future.
